# Reduced electron recombination of dye-sensitized solar cells based on TiO_2_ spheres consisting of ultrathin nanosheets with [001] facet exposed

**DOI:** 10.3762/bjnano.3.44

**Published:** 2012-05-07

**Authors:** Hongxia Wang, Meinan Liu, Cheng Yan, John Bell

**Affiliations:** 1 School of Chemistry, Physics and Mechanical Engineering, Queensland University of Technology, 2 George Street, GPO Box 2434, Brisbane, QLD 4001, Australia

**Keywords:** dye-sensitized solar cells, electrochemical impedance spectroscopy, electron recombination, TiO_2_ [001] facet, ultrathin nanosheets

## Abstract

An anatase TiO_2_ material with hierarchically structured spheres consisting of ultrathin nanosheets with 100% of the [001] facet exposed was employed to fabricate dye-sensitized solar cells (DSCs). Investigation of the electron transport and back reaction of the DSCs by electrochemical impedance spectroscopy showed that the spheres had a threefold lower electron recombination rate compared to the conventional TiO_2_ nanoparticles. In contrast, the effective electron diffusion coefficient, *D*_n_, was not sensitive to the variation of the TiO_2_ morphology. The TiO_2_ spheres showed the same *D*_n_ as that of the nanoparticles. The influence of TiCl_4_ post-treatment on the conduction band of the TiO_2_ spheres and on the kinetics of electron transport and back reactions was also investigated. It was found that the TiCl_4_ post-treatment caused a downward shift of the TiO_2_ conduction band edge by 30 meV. Meanwhile, a fourfold increase of the effective electron lifetime of the DSC was also observed after TiCl_4_ treatment. The synergistic effect of the variation of the TiO_2_ conduction band and the electron recombination determined the open-circuit voltage of the DSC.

## Introduction

In the past two decades, dye-sensitized solar cells (DSCs) have received substantial attention from both academic and industrial communities as one of the most promising low-cost, high-efficiency third-generation photovoltaic devices [1–2]. A typical DSC consists of a dye-coated TiO_2_ electrode, which is deposited on a fluorine-doped tin oxide (FTO) conductive-glass substrate, a I^−^/I_3_^−^ redox-couple-based electrolyte and a platinum counter electrode. Upon illumination, a photon with high energy (higher than the energy difference between the HOMO and LUMO level of the dye molecule) excites an electron from the ground state of the dye molecule to its excited state. The electron is then injected to the conduction band of the adjacent TiO_2_ material, owing to a favorable alignment of the energetics. The electron goes through a series of trapping/detrapping process in the TiO_2_ film before reaching the current collector, which is based on the conductive fluorine-doped tin oxide (FTO) substrate. Meanwhile, a parallel reaction, which involves transfer of the hole from the oxidized state of the dye (dye^+^) to the surrounding I^−^ ions of the redox couple of the electrolyte, occurs to regenerate the dye molecule, resulting in the formation of I_3_^−^ ions. The electrical circuit is completed through transfer of the electron, which arrives at the Pt counter electrode through the external circuit, to the I_3_^−^ ions of the electrolyte.

Apparently, the operation of a DSC depends on several reactions that occur at the interface between different materials [3]. In particular, the process of electron injection at the TiO_2_/dye interface and the electron recombination reaction at the TiO_2_/dye/electrolyte interface are critical because they control both the short-circuit current and open-circuit voltage of the DSC. The surface properties of the TiO_2_ material play an important role in both processes. The process of electron injection in DSCs is controlled by the energy difference between the conduction band of the TiO_2_ material and the LUMO level of the dye, and the process of electron recombination is mainly dominated by the interaction between the electron at the surface of TiO_2_ and I_3_^−^ ions in the electrolyte. Generally, the TiO_2_ used in DSCs is based on the anatase phase with the [101] facet exposed, due to the robust stability of this surface compared to other crystal facets [4]. It has been reported that the average surface energies of the different facets of anatase TiO_2_ lie in the order of [001] (0.90 J/m^2^) > [100] (0.53 J/m^2^) > [101] (0.44 J/m^2^) [5]. Apparently, the lowest surface energy of the [101] facet is the most stable surface of the TiO_2_ material. However, with the progress in synthesis techniques, other active facets of TiO_2_ crystals, such as [001], which is normally unstable due to a higher surface energy, can now be made [6]. In practice, TiO_2_ material with a large percentage of [001] high-energy surface has shown superior performance in applications such as water splitting and lithium-ion batteries [6–8]. Further investigation shows that the [001] surface is more beneficial to the photooxidization process through the O^−^ centers compared to the [101] surface, which contains more Ti^3+^ centers [9].

The different surface properties of the [001] and [101] facets of TiO_2_ are expected to have a profound effect on the chemicophysical processes in DSCs as well. Fan et al. reported that the [001] surface can absorb more dye molecules compared to the [101] surface [10]. However, the influence of the TiO_2_ [001] facet on the kinetics of electron transfer and back reaction has not been reported. A deep understanding of the role of the TiO_2_ [001] facet in these key processes of electron transport and recombination of DSCs is of great importance for both practical applications and basic research.

In this work, anatase TiO_2_ spheres with a hierarchical structure consisting of ultrathin nanosheets with 100% of the [001] facet exposed were synthesized and applied in dye-sensitized solar cells (DSCs). The photovoltaic performance of the DSCs with different concentrations of the hierarchically structured TiO_2_ spheres was evaluated. The kinetics of electron transport and back reaction of the DSCs with the spheres were investigated by electrochemical impedance spectroscopy. In addition, the effect of treatment by an aqueous solution of TiCl_4_ on the performance of the DSCs with the TiO_2_ spheres was discussed.

## Experimental

### Synthesis of TiO_2_ nanosheet particles

Hierarchically structured TiO_2_ spheres of the nanosheets were synthesized by following the method originally reported by Chen et al. [6]. Briefly, a precursor solution containing titanium isopropoxide (Sigma-Aldrich; 1.15 mL) and diethylenetriamine (DETA; 0.02 mL) in 32 mL isopropanol was prepared by vigorous magnetic stirring of the mixture of the three components at room temperature. The precursor solution was then transferred to a Teflon-lined stainless steel autoclave (45 mL volume, Parr Instrument Co.) for the hydrothermal reaction. The hydrothermal process was carried out at 200 °C for 24 h in an electric oven. After that, the autoclave was allowed to cool to room temperature naturally. The as-collected white powder was washed with deionized water and then ethanol several times to remove the organic residues. The powder was then dried at 80 °C for 5 h and finally sintered at 400 °C for 3 h to improve the crystallinity.

### Fabrication of TiO_2_ paste

TiO_2_ pastes for the DSCs were fabricated by mixing methylcellulose (*M*_w_ = 20,000), α-terpinol and the as-prepared TiO_2_ powder in a controlled amount, by using magnetic stirring at 80 °C for 48 h. Two sets of paste with different concentrations of the TiO_2_ powder were prepared. Paste A contained 13 wt % TiO_2_ powder, 2.6 wt % methylcellulose and 84.4 wt % α-terpinol. Paste B contained 25 wt % TiO_2_ powder, 2.5 wt % methylcellulose and 72.5 wt % α-terpinol. A commercial TiO_2_ paste (DSL-18-NR, Dyesol) consisting of anatase TiO_2_ nanoparticles with an average size of 20 nm was employed for comparison.

### Assembly of dye-sensitized solar cells

The procedure for the fabrication of the dye-sensitized solar cells was reported in our previous work [11–12]. Briefly, a substrate based on fluorine-doped tin oxide (FTO) conductive glass (TEC15, Pilkington) was thoroughly washed with detergent water, distilled water, acetone, isopropanol and ethanol in sequence under sonication for 15 min. The cleaned FTO substrate was first coated with a compact layer of TiO_2_ film by spray pyrolysis to reduce the electron back reaction at the interface between the bare FTO and the electrolyte. The substrate was then deposited with the as-prepared TiO_2_ paste or the commercial paste by a doctor-blading method using adhesive tape as a spacer to control the thickness of the film. The TiO_2_ film was dried on a hotplate at 90 °C for 10 min before being sintered at 450 °C for 30 min to form a mesoporous structure. The average thickness of the TiO_2_ film was 13 μm. TiCl_4_ post-treatment of the TiO_2_ film was carried out by immersing the sintered film in TiCl_4_ aqueous solution (40 mM) at 70 °C for 30 min. The film was washed with distilled water thoroughly and blow dried with N_2_ gas. The film was then resintered at 450 °C for 30 min. The geometrical area of the TiO_2_ film was 0.25 cm^2^.

The TiO_2_ film (with or without TiCl_4_ treatment) when it was still warm (around 80 °C) was immersed in a dye solution containing 0.25 mM *cis-*bis(isothiocyanato) bis(2,2´- bipyridyl-4,4´-dicarboxylate) ruthenium(II) bis-tetrabutylammonium (N719, Dyesol) in ethanol for 16 h to form a dye-coated photoanode. A platinum counter electrode was prepared by dropping 5 μL isopropanol solution containing H_2_PtCl_6_·6H_2_O (5 mM) onto a cleaned FTO substrate (1.5 × 1.0 cm^2^). After being allowed to dry in air, the substrate was then sintered at 390 °C for 15 min in an electric oven to form a thin Pt layer on the FTO substrate. A dye-sensitized solar cell was assembled by sealing the dye-coated TiO_2_ electrode with the platinum-coated FTO counter electrode by using a thermal plastic (Surlyn 1705) at 130 °C. The electrolyte composed of 0.6 M 1-propyl-3-methylimidiazolium iodide, 0.05 M I_2_, 0.1 M guanidinium thiocyanate, 0.2 M NaI, 0.1 M *N*-methyl benzimidazole in 3-methoxypropionitrile was introduced into the space between the electrodes through the holes that were predrilled in the Pt counter electrode by a vacuum-assisted technique. The holes were then sealed by using a Surlyn film covered with a microscope slip.

### Characterization

The morphology and the crystal structure of the as-prepared TiO_2_ powder were investigated by scanning electron microscope (SEM, FEI Quanta 200) and powder X-ray diffraction (XRD, PANanalytical Xpert Pro), respectively. Transmission electron microscopy (TEM, Philips CM 200) was used to monitor the detailed structure of the TiO_2_ powder. The thickness of the TiO_2_ films for the DSCs was determined by a profilometer (Dektak 150). The photocurrent density–voltage (*J*–*V*) characteristics of the DSCs were obtained by using a Xe lamp (150 W) based solar simulator (Newport), by recording the current produced by the cells as a function of the applied bias under AM1.5 illumination (100 mW/cm^2^) with a computer-controlled digital source meter (Keithley 2420). The illumination intensity of the incident light from the solar simulator was measured with a silicon photodiode, which was calibrated with an optical meter (1918-C, Oriel). Aluminum foil with a size comparable to the active area of the TiO_2_ film was used as a reflector on the counter electrode side of the DSCs during the *J–V* measurement.

The electrochemical impedance spectroscopy (EIS) of the DSCs was measured in the frequency range of 50,000–0.1 Hz at room temperature by a Versa-stat 3 electrochemical workstation (Princeton Applied Research). The EIS measurement was carried out under illumination, which was provided by a light emitting diode (LED, 627 nm) at open-circuit. The intensity of the incident illumination on the front side of the DSC (TiO_2_ side) was adjusted by using a combination of neutral density filters. The EIS spectrum was analyzed with a Zview software, by using a transmission-line-based equivalent circuit to obtain the information of chemical capacitance, electron-recombination resistance and electron-transport resistance of the DSCs [12–13].

## Results and Discussion

**Figure 1:**
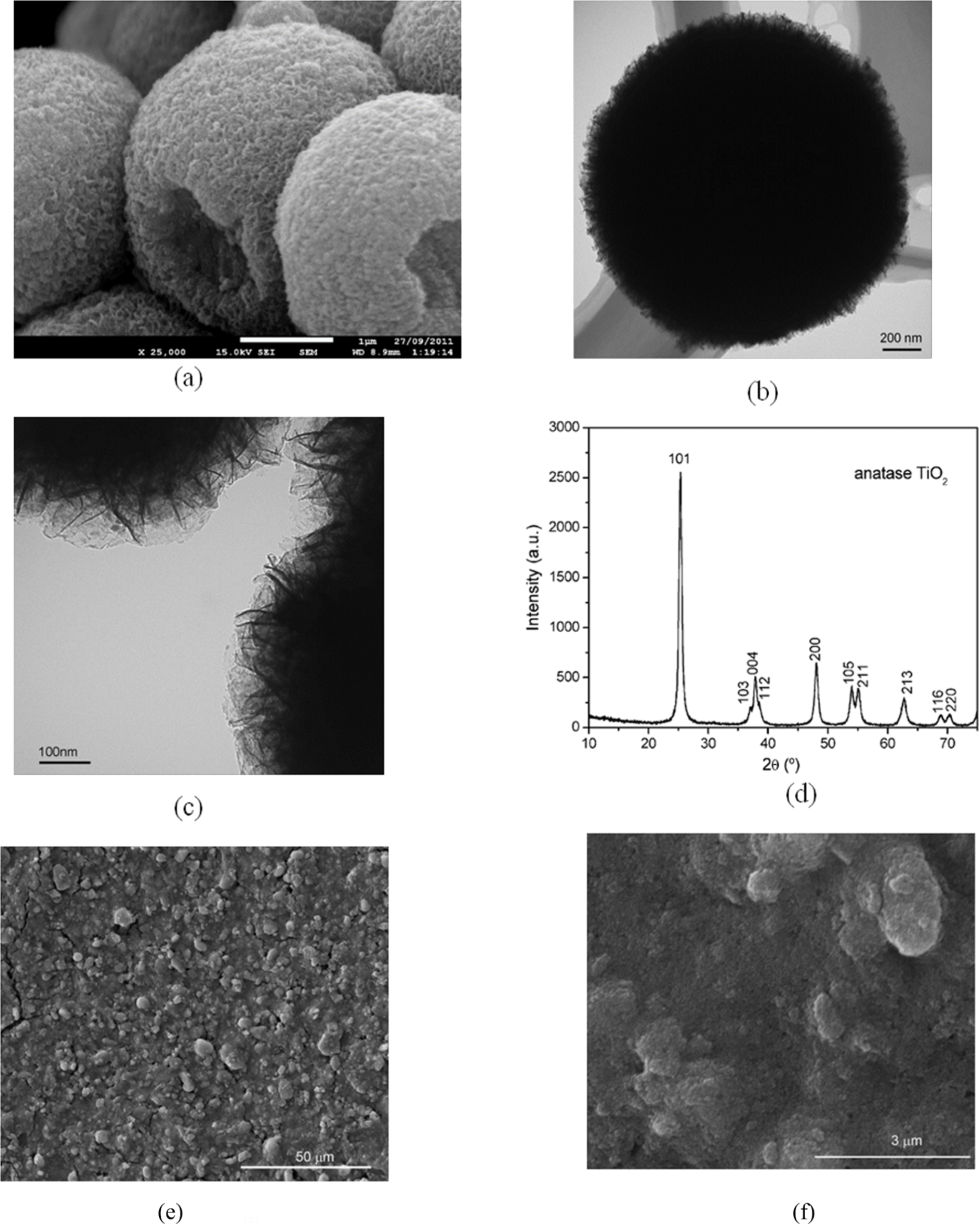
Images of TiO_2_ particles by SEM (a) and by TEM (b, c) as well as XRD pattern of the TiO_2_ particles (d) and morphology of the TiO_2_ film consisting of the as-prepared sphere particles (e, f).

Figure 1a shows the image of the as-prepared TiO_2_ powder by SEM. The material consists of microsized particles with spherical shape. The surface of the sphere is very rough and appears fluffy. The diameter of the sphere is around 1.6 μm as determined by TEM (Figure 1b). TEM images (Figure 1b, Figure 1c) also illustrate that the sphere has a substructure, which consists of ultrathin nanosheets packed together. It is speculated that the sphere is formed through self-assembly of the nanosheets to realize a minimum surface energy. Some spheres have pits on the surface, which may be due to the insufficient reaction duration. The measurement of the N_2_ adsorption/desorption isotherms of the TiO_2_ powder shows that the specific surface area of the TiO_2_ spheres is 82 m^2^/g, which is slightly higher than the specific surface area of the film made from the commercial TiO_2_ paste (DSL-18NR, Dyesol. Surface area: 72.9 m^2^/g) [14]. The large surface area of the material suggests that the nanosheets are probably loosely packed such that a greater surface area is exposed. The XRD pattern of the material (Figure 1d) shows that the as-prepared TiO_2_ powder is anatase with a tetragonal structure and space group *I*4_1_*/amd* (JCPDS card, No. 71-1169). Both the TEM images and the XRD results are in good agreement with the results reported by Chen et al. According to Chen et al., the TiO_2_ spheres synthesized by this method have 100% of the [001] surface exposed [6].

The SEM image of the TiO_2_ film consisting of the spheres is shown in Figure 1e and Figure 1f. Apparently, the TiO_2_ particles are connected to each other in the film. Figure 1f shows that the film contains a large number of small pores. However, the sphere of the TiO_2_ particles is rarely seen in the film. This indicates that the mechanical force of grinding and sonication employed in the preparation of the film broke up the spheres into small particles, probably in the form of nanosheets. Nevertheless, the same XRD pattern of the sintered TiO_2_ film (not shown) indicates that the film has the same surface properties as the spheres.

### *J*–*V* characteristics of the DSCs

**Figure 2:**
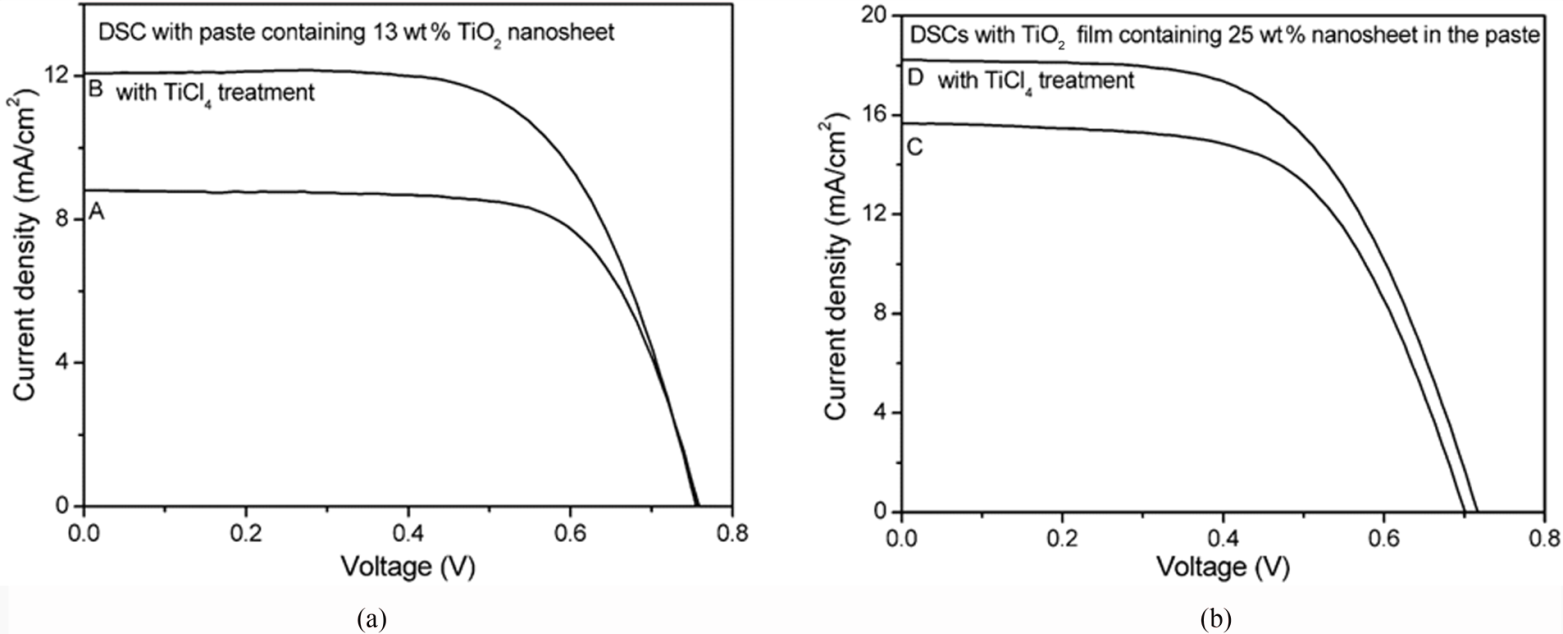
*J–V* characteristics of the dye-sensitized solar cells made from paste A containing 13 wt % TiO_2_ spheres (a) and from paste B containing 25 wt % spheres (b) with (curve B and D) and without (curve A and C) TiCl_4_ treatment.

The *J*–*V* characteristics of the DSCs with the TiO_2_ film made from paste A, which contained 13 wt % TiO_2_ spheres with and without TiCl_4_ post-treatment, is shown in Figure 2a. The DSC solely based on paste A without TiCl_4_ treatment (curve A) produced a short-circuit current density (*J*_sc_) of 8.79 mA/cm^2^ and open-circuit voltage (*V*_oc_) of 0.76 V. In contrast, when the TiO_2_ film was subjected to TiCl_4_-solution treatment, the *J*_sc_ of the DSC (curve B) increased to 12.1 mA/cm^2^, which is 37.5% higher than that of curve A.

Figure 2b shows the *J–V* performance of the DSCs made from paste B, which contained 25 wt % as-prepared TiO_2_ nanosheet-based particles. The *J*_sc_ of the cell was 15.6 mA/cm^2^ and *V*_oc_ = 0.70 V (Figure 2b, curve C) when there was no TiCl_4_ treatment. Compared to curve A, it was found that the ratio of the *J*_sc_ of curve C to that of curve A ((15.6 mA/cm^2^)/(12.1 mA/cm^2^) = 1.77) is very close to the ratio of the concentration of the TiO_2_ spheres in the two pastes ((TiO_2_ wt % in paste B)/(TiO_2_ wt % in paste A) = 25/13 = 1.92). This suggests that the higher *J*_sc_ of the DSC made from paste B is due to the availability of more TiO_2_ particles in the film, which can absorb more dye molecules, leading to a stronger light absorption. The *J*_sc_ of the DSC made from paste B was further increased from 15.6 mA/cm^2^ (Figure 2b, curve C) to 18.2 mA/cm^2^ when the TiO_2_ film was processed with TiCl_4_ solution (Figure 2b, curve D). Meanwhile, the *V*_oc_ of curve D was 20 mV higher than that of curve C, suggesting a beneficial effect of the TiCl_4_ post-treatment on *V*_oc_ as well. The best performance was obtained in the case of curve D with power conversion efficiency = 7.57% (Figure 2b), which is comparable to the efficiency (η = 7.52%) of the DSCs made from the commercial paste (*I*–*V* curve is not shown). The detailed characteristic parameters of the performance of the DSCs with different TiO_2_ pastes are shown in Table 1.

**Table 1:** Characteristic performance parameters of the dye-sensitized solar cells.

cell name	*J*_sc_ (mA/cm^2^)	*V*_oc_ (V)	*FF*	efficiency (%)

13 wt % TiO_2_ nanosheet (curve A)	8.79	0.757	0.700	4.66
13 wt % TiO_2_ nanosheet with TiCl_4_ treatment (curve B)	12.07	0.754	0.646	5.88
25 wt % TiO_2_ nanosheet (curve C)	15.60	0.700	0.610	6.66
25 wt % TiO_2_ nanosheet with TiCl_4_ treatment (curve D)	18.20	0.720	0.580	7.57
DSC made from TiO_2_ nanoparticles	16.50	0.755	0.604	7.52

### Electrochemical impedance spectroscopy

**Figure 3:**
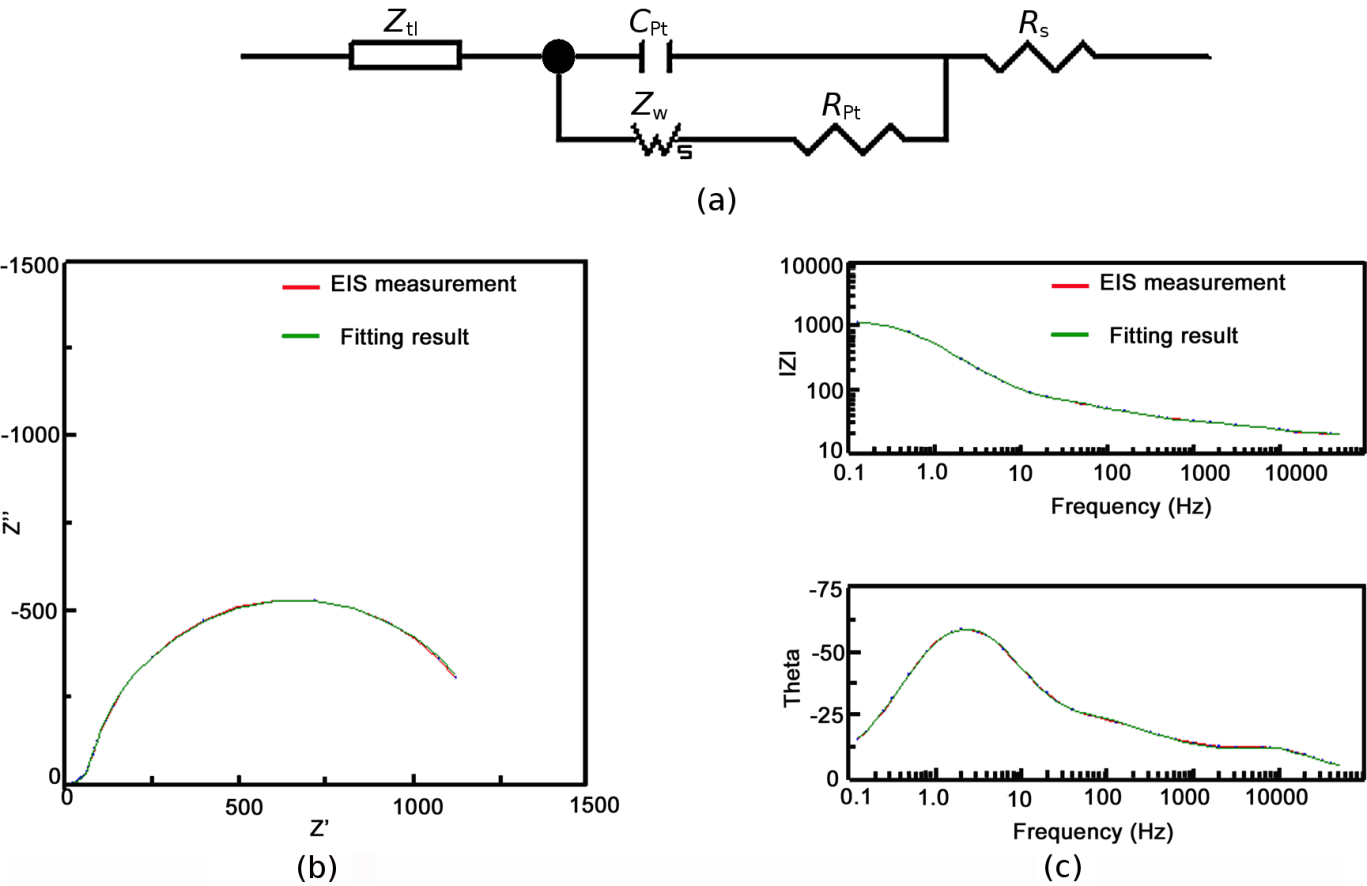
Equivalent circuit (a) and the Nyquist plot (b) and Bode plot (c) of the impedance spectrum of a dye-sensitized solar cell.

Information on the charge-transfer and charge-transport process in DSCs can be measured by small-perturbation-based transient methods, such as electrochemical impedance spectroscopy (EIS) or intensity modulated photocurrent spectroscopy (IMPS) and intensity modulated photovoltage spectroscopy (IMVS) [13,15]. Compared to IMPS and IMVS, the advantage of the EIS method for characterization of DSCs lies in the fact that both the effective electron lifetime, τ_n_, and the effective electron diffusion coefficient, *D*_n_, can be obtained in one measurement. This is achieved by fitting the EIS spectrum using a suitable equivalent circuit that mimics the physical process in the device. The equivalent circuit that depicts the process of electron trapping/detrapping in DSCs is shown in Figure 3a. It contains a series resistance, *R*_s_, a capacitance at the Pt electrode/electrolyte interface, *C*_Pt_, and a resistance for the charge-transfer process between electrons at the Pt electrode and I_3_^−^ ions of the electrolyte, *R*_Pt_. *Z*_w_ is the Warburg resistance arising from the ion transport in the electrolyte and *Z*_tl_ is a distribution line describing the electron transport and recombination in the mesoporous TiO_2_ film [13,16]. A typical EIS spectrum of a DSC is shown in Figure 3b for the Nyquist plot and Figure 3c for the Bode plot. The corresponding fitting results (green line) using the equivalent circuit are also shown in Figure 3b and Figure 3c. The distorted semicircle in the high frequency range (above 10 Hz) is ascribed to the electron transfer process at the interface of Pt counter electrode/electrolyte combined with the electron-transport process in the TiO_2_ film (the semicircle corresponding to the electron transport process in TiO_2_ is buried in the semicircle of the charge-transfer process at the Pt/electrolyte interface in the spectrum) [3]. The large semicircle in the lower frequency range (10–0.1 Hz) is due to the electron recombination process in the TiO_2_ film. Under a high incident illumination intensity, the density of the photogenerated electron in the TiO_2_ film is very high (up to 10^18^/cm^3^) and the TiO_2_ film becomes conductive [17]. In this case, the resistance corresponding to the electron-transport process becomes too small to be observed in the EIS spectrum. Consequently, the EIS spectrum is mainly dominated by the electron recombination process. Nevertheless, under low incident illumination intensity, the conductivity of the TiO_2_ film is very low due to a low density of photogenerated electrons. In this case, the main feature of the EIS spectrum is due to the transport of electrons in the TiO_2_ film. Hence, an accurate fitting of the EIS spectrum of a DSC using the equivalent circuit is normally obtained in the illumination range in which both the electron-transport resistance and the electron-recombination resistance are substantial [13]. Only the results of good fits are shown in this work.

### Comparison of electron transport and recombination of the DSC based on TiO_2_ spheres and nanoparticles

The electron-recombination process in DSCs is reflected by the effective electron lifetime, τ_n_, whereas the electron-transport process is manifested by the effective electron diffusion coefficient *D*_n_. Bisquert et al. showed that both τ_n_ and *D*_n_ of a DSC are dependent on the distribution of the density of electrons in the conduction band (free electron) and in the trap states (trapped electron) of the TiO_2_ film as well as the lifetime and diffusion coefficient of free electron (τ_0_ and *D*_0_), through the following consideration:

[1]
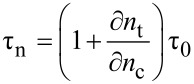


and

[2]
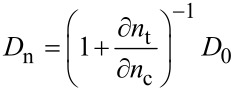


where *n*_t_ and *n*_c_ are the densities of the trapped electron and free electron, respectively [18].

The charge distribution, *g*(*E*), in a mesoporous TiO_2_ film is described by [18–19]:

[3]
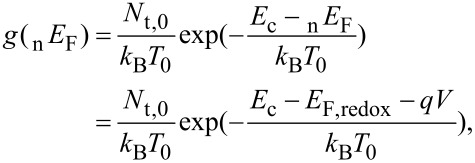


where _n_*E*_F_ is the quasi Fermi level of TiO_2_, *E*_c_ the conduction band of TiO_2_, *E*_F_*_,_*_redox_ the potential energy of the redox couple, *N*_t,0_ the total density of the trapped electrons, *k*_B_ is the Boltzmann constant and *T*_0_ the characteristic temperature that reflects the profile of the charge distribution in TiO_2_.

Therefore, comparison of the change of τ_n_ and *D*_n_ in DSCs due to the different material composition should be made by using the density of charge as the reference, provided that the distribution profile of charge density is the same [20].

The density of charge in the TiO_2_ film is reflected by the chemical capacitance, *C*_μ_, which is measured by EIS, through the relationship [18]:

[4]
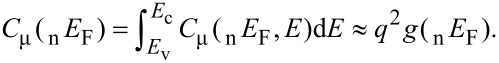


where *E*_v_ is the valence band of TiO_2_. Thus, we employ the density of chemical capacitance as a reference for the investigation of the variation of τ_n_ and *D*_n_ in the following.

**Figure 4:**
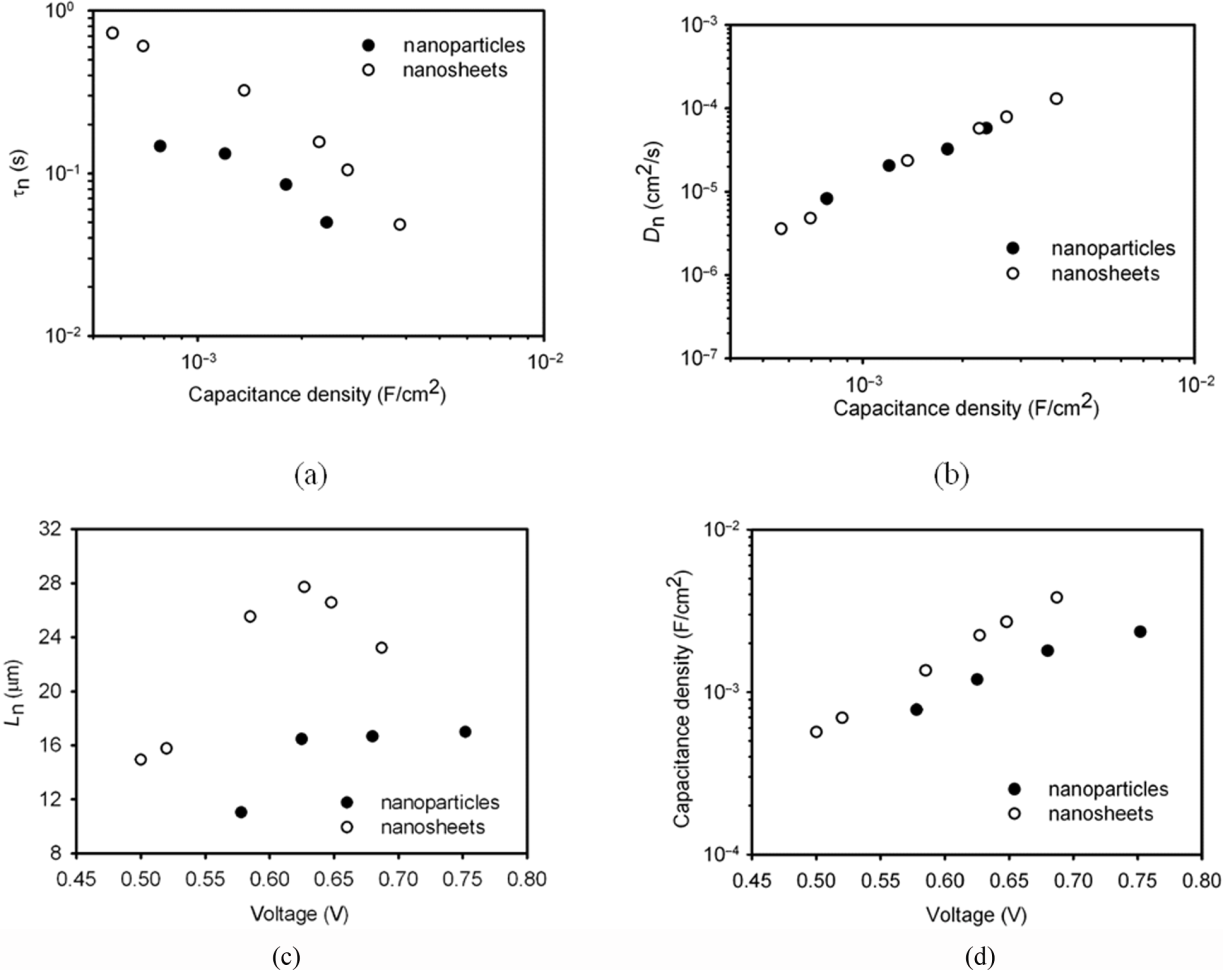
Comparison of the effective electron lifetime, τ_n_, (a) and effective electron diffusion coefficient, *D*_n_, (b) and electron diffusion length, *L*_n_, (c) of the DSCs with TiO_2_ nanosheets (open circle) and with conventional nanoparticles (solid circle). (d) Variation of the chemical capacitance density of the nanosheets and nanoparticles as a function of voltage.

Figure 4a shows the τ_n_ of the DSCs with the TiO_2_ films consisting of the nanosheet-based spheres and the conventional nanoparticles as a function of the chemical capacitance density. It is found the τ_n_ of the nanosheets based DSC is nearly threefold higher than that of the nanoparticles for a constant capacitance density. This suggests that the TiO_2_ film with the spheres has a lower electron-recombination reaction rate compared to the film with the nanoparticles. Besides τ_n,_ the effective electron diffusion coefficient, *D*_n_, is another important parameter that determines the performance of a DSC. The comparison of the *D*_n_ of the cell based on the spheres and the nanoparticles is shown in Figure 4b. It is interesting that both materials show the same *D*_n_, suggesting that the electron transport is not affected by the morphology and the exposed crystal facet of the TiO_2_ material. The identical *D*_n_ also suggests that the diffusion coefficient of the free electron is the same for the two materials, according to Equation 2 [13]. It also justifies the assumption that the profile of the distribution of charge density is the same in the two types of TiO_2_ film. In contrast, the different τ_n_ suggests that the free-electron lifetime of the spheres is different to that of the nanoparticles. The high τ_n_ of the spheres could be related to the properties of the [001] facet, but clarification of this issue requires further investigation. As a consequence, the electron diffusion length, *L*_n_, which depends on both the τ_n_ and *D*_n_ by 
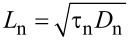
, is up to 1.6-fold higher for the nanosheet-based TiO_2_ spheres compared to that of the nanoparticles (Figure 4c). It is found that the *L*_n_ of the DSC based on the nanoparticles is only around 16 μm (Figure 4c), which is comparable to the thickness of the TiO_2_ film (13 μm). A previous study has shown that the *L*_n_ of a DSC needs to be at least three times the thickness of the TiO_2_ film in order to collect most of the photogenerated electrons [13]. Therefore, the short *L*_n_ may limit the performance of the DSC. The higher *L*_n_ of the spheres-based DSC should lead to a higher electron collection efficiency compared to its nanoparticles counterpart.

Besides *J*_sc_, *V*_oc_ is another key performance parameter of a DSC. The maximum voltage of a DSC is determined by the potential difference between the conduction band of TiO_2_ and the redox potential of I^−^/I_3_^−^ in the electrolyte. Obviously, the position of the TiO_2_ conduction band edge, *E*_c_, has a direct impact on the open-circuit voltage (*V*_oc_) of the DSC. Thus, it is important to know the relative position of the *E*_c_ of the nanosheet-based spheres relative to the nanoparticles in order to determine the reason for the different *V*_oc_. According to Equation 3, the change of *E*_c_ of TiO_2_ can be monitored by the variation of the voltage (*V*) of the DSC at a constant electron density.

As shown in Figure 4d, the *E*_c_ of the nanosheet-based spheres is found to be 100 meV lower than that of the nanoparticles. The lower *E*_c_ of the spheres is probably due to the different dye loading on the TiO_2_ films. According to Fan et al. [10], the TiO_2_ [001] facet can absorb more dye than the [101] facet. Nazeeruddin et al. confirmed that the dye molecule is adsorbed on TiO_2_ particles mainly through the carboxylic acid group (–COOH) [4], leading to the protonation of the surface of TiO_2_ and the downward shift of the *E*_c_. The more dye molecules are adsorbed on the TiO_2_ film, the more downward shift is expected for the *E*_c_.

### Effect of TiCl_4_ treatment

The strategy of treating TiO_2_ mesoporous films with TiCl_4_ aqueous solution has been extensively employed to improve the performance of DSCs. In most cases, it is found that the *J*_sc_ of the DSC is enhanced, while the *V*_oc_ is reduced after the TiCl_4_ treatment of the film. O’Regan et al. found that TiCl_4_ treatment caused 80 meV downward shift of the TiO_2_ conduction band, resulting in an increased driving force for the electron-injection process. They reported that the enhanced *J*_sc_ was owing to an improved electron-injection efficiency of the DSC [21–22]. In the following section, the influence of the TiCl_4_ solution treatment on the *E*_c_ of the TiO_2_-spheres-based film and on the kinetics of electron transport and back reaction of the corresponding DSCs is investigated.

**Figure 5:**
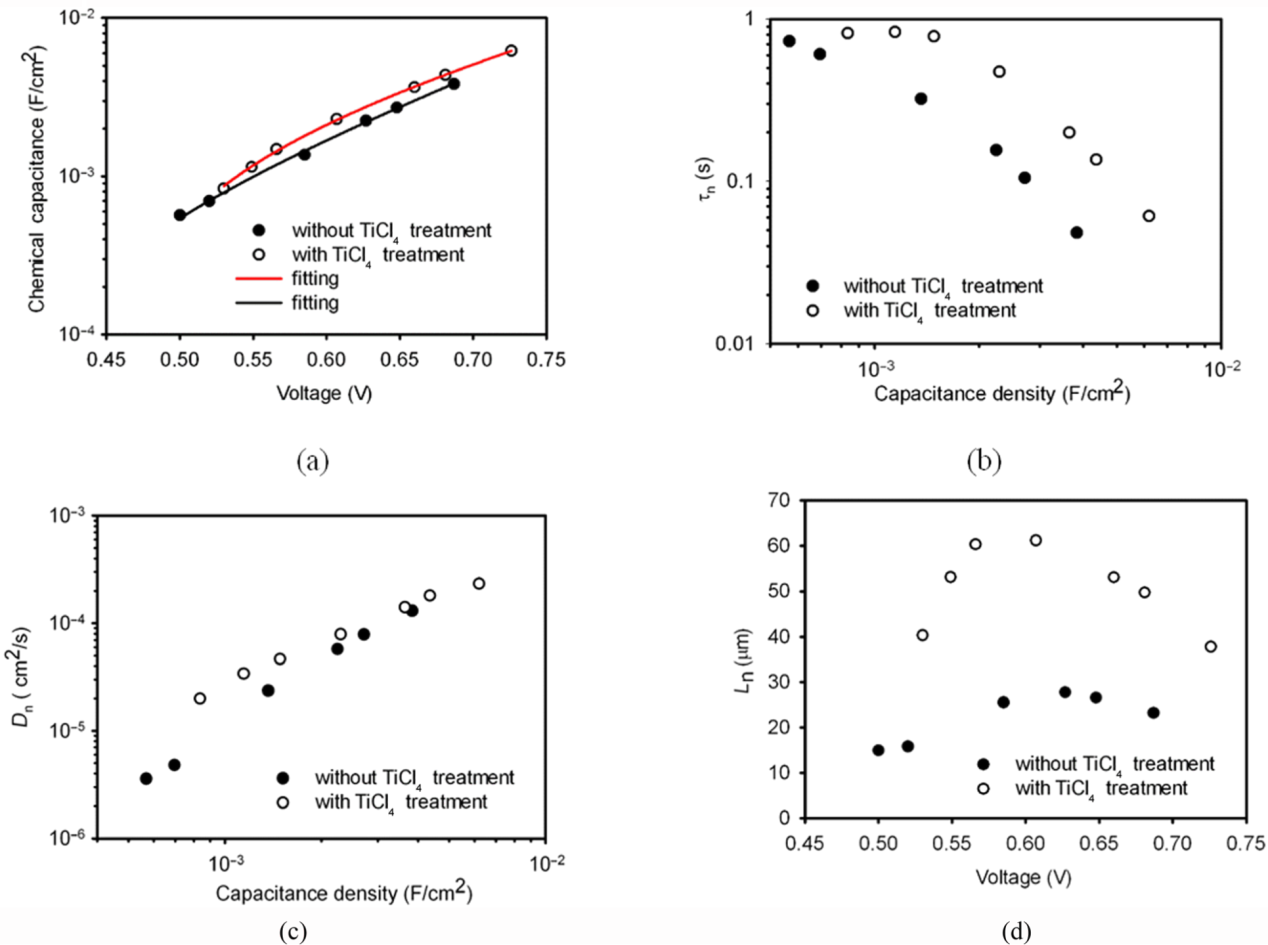
Comparison of dye-sensitized solar cells based on hierarchically structured TiO_2_ spheres with and without TiCl_4_ post-treatment. (a) Chemical capacitance vs voltage; (b) effective electron lifetime, τ_n_, as a function of density of chemical capacitance; (c) effective electron diffusion coefficient, *D*_n_, as a function of density of chemical capacitance; and (d) electron diffusion length, *L*_n_ as a function of voltage.

Figure 5a illustrates the chemical capacitance density of the DSCs made from paste B with and without TiCl_4_ treatment, as a function of the voltage. It is found that, at a constant charge density, the voltage of the cell with TiCl_4_ treatment is lower than that of the DSC without the treatment. The maximum difference in voltage between the cells is around 30 mV. Provided that the distribution profile of the charge density is the same for the TiO_2_ film with and without TiCl_4_ treatment, the reduced potential of the DSC with TiCl_4_ treatment means that the TiCl_4_ treatment caused a downward shift of the TiO_2_ conduction band by 30 meV, which may decrease the maximum voltage the DSC can achieve. This observation is in good agreement with the results reported by O’Regan et al. [21]. However, the *V*_oc_ of the cell with TiCl_4_ treatment is actually 20 mV higher than the DSC without the treatment, as shown in Figure 2b. This indicates that the electron recombination of the DSC is probably affected by the TiCl_4_ treatment.

Figure 5b shows the τ_n_ as a function of capacitance density of the DSC with and without TiCl_4_ treatment. It is found that τ_n_ is enhanced by a factor of 3.8 after TiCl_4_ treatment. In contrast, *D*_n_ of the DSCs is relatively unchanged with the TiCl_4_ treatment (Figure 5c). Owing to the enhanced τ_n_, the electron diffusion length, *L*_n_, of the DSC is enhanced by a factor of two through TiCl_4_ treatment (Figure 5d). Hence, the improved voltage (20 mV) of the DSC (Figure 2b, curve D) with TiCl_4_ treatment compared to the cell without TiCl_4_ treatment (curve C in Figure 2b) should be a result of a synergistic effect of the decreased TiO_2_ conduction band and the increased electron lifetime. Apparently, the beneficial effect of the enhanced electron lifetime on *V*_oc_ surpasses the negative effect of the downward shift of the *E*_c_ of TiO_2_, leading to a higher *V*_oc_.

## Conclusion

Dye-sensitized solar cells with a TiO_2_ electrode made from hierarchically structured TiO_2_ spheres, consisting of nanosheets with 100% of the [001] facet exposed, were assembled and characterized in terms of the device performance, the kinetics of electron transport and back reaction. It was found that the TiO_2_-spheres-based DSCs generated an energy conversion efficiency of 7.57%, which is comparable to the conventional TiO_2_ nanoparticles. Investigation of the kinetics of electron transport and back reaction of the DSCs showed that the spheres had a threefold higher effective electron lifetime compared to the nanoparticles. However, the effective electron diffusion coefficient, *D*_n_, of the DSCs was not affected by the different morphology and exposed crystal facets of the TiO_2_ material. Monitoring of the variation of the conduction band of the dyed TiO_2_ film disclosed that the *E*_c_ of the spheres-based TiO_2_ electrode was 100 meV lower than that of the nanoparticles.

This work also investigated the influence of treatment with TiCl_4_ aqueous solution on the *E*_c_ of the TiO_2_ spheres and on the τ_n_ and *D*_n_ of the corresponding DSCs. It was found that TiCl_4_ treatment caused a downward shift (30 meV) of the TiO_2_ conduction band and a fourfold increase of the τ_n_, whereas the *D*_n_ of the cell was not significantly affected by the TiCl_4_ treatment.
